# Ventricular volume asymmetry as a novel imaging biomarker for disease discrimination and outcome prediction

**DOI:** 10.1093/ehjopen/oeae059

**Published:** 2024-07-25

**Authors:** Celeste McCracken, Liliana Szabo, Zaid A Abdulelah, Dorina-Gabriela Condurache, Hajnalka Vago, Thomas E Nichols, Steffen E Petersen, Stefan Neubauer, Zahra Raisi-Estabragh

**Affiliations:** Division of Cardiovascular Medicine, Oxford Centre for Clinical Magnetic Resonance Research, Radcliffe Department of Medicine, University of Oxford, National Institute for Health Research Oxford Biomedical Research Centre, Oxford University Hospitals NHS Foundation Trust, Oxford OX3 9DU, UK; Barts Heart Centre, St Bartholomew’s Hospital, Barts Health NHS Trust, West Smithfield, London EC1A 7BE, UK; William Harvey Research Institute, NIHR Barts Biomedical Research Centre, Queen Mary University of London, Charterhouse Square, London EC1M 6BQ, UK; Heart and Vascular Center, Semmelweis University, Budapest 1122, Hungary; Barts Heart Centre, St Bartholomew’s Hospital, Barts Health NHS Trust, West Smithfield, London EC1A 7BE, UK; Barts Heart Centre, St Bartholomew’s Hospital, Barts Health NHS Trust, West Smithfield, London EC1A 7BE, UK; William Harvey Research Institute, NIHR Barts Biomedical Research Centre, Queen Mary University of London, Charterhouse Square, London EC1M 6BQ, UK; Heart and Vascular Center, Semmelweis University, Budapest 1122, Hungary; Department of Sports Medicine, Semmelweis University, Budapest 1085, Hungary; Wellcome Centre for Integrative Neuroimaging (WIN FMRIB), University of Oxford, Oxford OX3 9DA, UK; Big Data Institute, University of Oxford, Oxford OX3 7LF, UK; Nuffield Department Population Health, Big Data Institute, University of Oxford, Oxford OX3 7LF, UK; Barts Heart Centre, St Bartholomew’s Hospital, Barts Health NHS Trust, West Smithfield, London EC1A 7BE, UK; William Harvey Research Institute, NIHR Barts Biomedical Research Centre, Queen Mary University of London, Charterhouse Square, London EC1M 6BQ, UK; Health Data Research UK, London NW1 2BE, UK; Division of Cardiovascular Medicine, Oxford Centre for Clinical Magnetic Resonance Research, Radcliffe Department of Medicine, University of Oxford, National Institute for Health Research Oxford Biomedical Research Centre, Oxford University Hospitals NHS Foundation Trust, Oxford OX3 9DU, UK; Barts Heart Centre, St Bartholomew’s Hospital, Barts Health NHS Trust, West Smithfield, London EC1A 7BE, UK; William Harvey Research Institute, NIHR Barts Biomedical Research Centre, Queen Mary University of London, Charterhouse Square, London EC1M 6BQ, UK

**Keywords:** Cardiovascular magnetic resonance, UK Biobank, Imaging biomarkers, Cardiovascular disease, Cardiovascular risk, Epidemiology

## Abstract

**Aims:**

Disruption of the predictable symmetry of the healthy heart may be an indicator of cardiovascular risk. This study defines the population distribution of ventricular asymmetry and its relationships across a range of prevalent and incident cardiorespiratory diseases.

**Methods and results:**

The analysis includes 44 796 UK Biobank participants (average age 64.1 ± 7.7 years; 51.9% women). Cardiovascular magnetic resonance (CMR) metrics were derived using previously validated automated pipelines. Ventricular asymmetry was expressed as the ratio of left and right ventricular (LV and RV) end-diastolic volumes. Clinical outcomes were defined through linked health records. Incident events were those occurring for the first time after imaging, longitudinally tracked over an average follow-up time of 4.75 ± 1.52 years. The normal range for ventricular symmetry was defined in a healthy subset. Participants with values outside the 5th-95th percentiles of the healthy distribution were classed as either LV dominant (LV/RV > 112%) or RV dominant (LV/RV < 80%) asymmetry. Associations of LV and RV dominant asymmetry with vascular risk factors, CMR features, and prevalent and incident cardiovascular diseases (CVDs) were examined using regression models, adjusting for vascular risk factors, prevalent diseases, and conventional CMR measures. Left ventricular dominance was linked to an array of pre-existing vascular risk factors and CVDs, and a two-fold increased risk of incident heart failure, non-ischaemic cardiomyopathies, and left-sided valvular disorders. Right ventricular dominance was associated with an elevated risk of all-cause mortality.

**Conclusion:**

Ventricular asymmetry has clinical utility for cardiovascular risk assessment, providing information that is incremental to traditional risk factors and conventional CMR metrics.

## Graphical Abstract

**Graphical Abstract:**
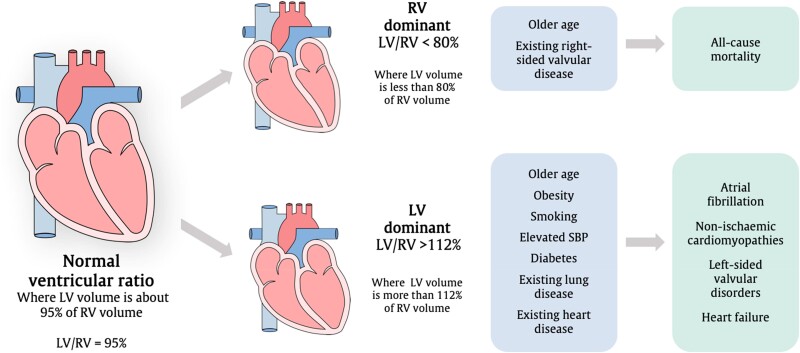

## Introduction

Cardiovascular imaging captures early ventricular remodelling indicative of pre-clinical disease and future cardiovascular risk.^1^ Left and right ventricular (LV and RV) volume measures are routinely used in clinical practice to distinguish health from disease, with dilated ventricles linked to various cardiomyopathies and volume overload states. More recent research suggests that the relative size of the ventricles, in addition to their absolute size, may indicate underlying disease processes.^2^

Most of the existing research into the importance of ventricular asymmetry is confined to highly select disease states, where right heart dilatation is prominent. For instance, RV/LV volume and diameter ratio have demonstrable value for risk stratification in pulmonary embolism,^3^ pulmonary hypertension,^4^ and congenital heart disease.^5^ Furthermore, the RV/LV ratio has been used to assess the progression of RV arrhythmogenic cardiomyopathy^2^ and to quantify the haemodynamic consequence of pulmonary valve regurgitation in patients with repaired Tetralogy of Fallot.^5^ The clinical utility of the ventricular ratio metric has not been evaluated outside of these very specific clinical contexts, and its utility in wider clinical and population cohorts is unknown. Given that this ratio may be readily calculated from routinely available metrics, its potential value as an imaging biomarker merits investigation.

Cardiovascular magnetic resonance (CMR) is the reference modality for volumetric ventricular quantification, providing superior endocardial border definition and highly reproducible biomarkers.^6,7^ The UK Biobank Imaging Study includes standardized CMR scans for a very large cohort of population-dwelling participants, linked to longitudinally tracked health outcomes, providing the ideal platform to evaluate the clinical utility of novel imaging biomarkers.

In this study of 44 796 UK Biobank participants, we (i) characterize the population distribution of ventricular asymmetry and define the normal range in a subset of healthy participants; (ii) evaluate associations of ventricular asymmetry with demographics, lifestyle factors, and CMR measures of atrial and ventricular structure and function; (iii) examine associations of ventricular asymmetry with a range of prevalent and incident cardiovascular diseases (CVDs) independent of vascular risk factors and other CMR metrics.

## Methods

### Study population

The UK Biobank recruited half a million participants from a range of urban and rural settings across the UK. Individuals aged 40–69 years living within 25 miles of 22 UK Biobank assessment centres were identified from National Health Service registers and included in the study between 2006 and 2010, where extensive baseline phenotyping was conducted.^8^ The UK Biobank Imaging Study aims to scan a 20% (*n* = 100 000) subset of the original participants using a standardized acquisition protocol for multi-organ imaging, which includes CMR.^9^ Health record linkage has been established for the entire UK Biobank cohort, permitting longitudinal tracking of incident health events. For this study, we included 44 796 participants with imaging metrics available, with scanning taking place between April 2014 and March 2020 (see Supplementary material online, *Figure S1*).

### Ethics

This study complies with the Declaration of Helsinki; the work was covered by the ethical approval for UK Biobank studies from the NHS National Research Ethics Service on 17 June 2011 (Ref. 11/NW/0382) and extended on 18 June 2021 (Ref. 21/NW/0157) with written informed consent obtained from all participants.

### Cardiovascular magnetic resonance protocol and image analysis

Cardiovascular magnetic resonance imaging was performed on 1.5 T scanners (MAGNETOM Aera, Syngo Platform VD13A, Siemens Healthcare, Erlangen, Germany) in dedicated imaging centres using standardized staff training and equipment.^10^ Image analysis was conducted with previously validated automated pipelines.^9,11–13^ This study included the following CMR metrics: LV end-diastolic volume index (LVEDVi), LV mass index (LVMi), maximal wall thickness (max WT), myocardial native T1, LV ejection fraction (LVEF), LV global function index (LVGFI), global longitudinal strain (GLS), global circumferential strain (GCS), RV end-diastolic volume index (RVEDVi), RV ejection fraction (RVEF), left atrial ejection fraction (LAEF), right atrial ejection fraction, and aortic distensibility (AoD).

### Definition of left ventricular and right ventricular dominant ventricular asymmetry

We calculated LV/RV ratios using LVEDVi and RVEDVi. To understand the normal variations of ventricular asymmetry, we created a subset of 37 200 participants free from clinically diagnosed cardiorespiratory disease at time of imaging (see Supplementary material online, *Figure S1*). This subset was used to define the average normal ventricular symmetry for men and women (*Table 1* and Supplementary material online, *Table S1*). We defined the 5th and 95th percentiles, indicating the range within which 90% of healthy individuals fall. Outside this range, we observed two types of ventricular asymmetry: cases where the LV is disproportionately larger than the RV (LV/RV > 112%), labelled as ‘LV dominant’, and cases where the RV is significantly larger relative to the LV (LV/RV < 80%), labelled as ‘RV dominant’. To simplify the associations with ventricular asymmetry, we created binary variables for abnormal ventricular ratios. Following an exploratory analysis, we added a third category for mildly asymmetric cases, where LV/RV is not abnormal, but is more than 1 standard deviation above the healthy mean. We call this category ‘mildly LV dominant’.

**Table 1:** Normal range of ventricular symmetry in a healthy subset (*n* = 32 700)

Ratio	Sex/group	Mean	SD	5th percentile	95th percentile	Mean + 1 SD cut-off
LV/RV ×100	Female	97	10	82	113	107
	Male	93	10	79	109	103
	Combined	95	10	80	112	**105**

The value in bold (105) indicates the mean + 1 SD cut-off for the combined group. A LV/RV ratio of 105 or below is considered within the normal range, while a ratio above 105 is considered ’mildly LV dominant’.

LV, left ventricular end-diastolic volume; RV, right ventricular end-diastolic volume; SD, standard deviation.

### Definition of covariates

Sex, ethnicity, family history of heart disease, and Townsend deprivation index were recorded at baseline recruitment. Age, body mass index (BMI), waist-to-hip ratio, and systolic blood pressure (SBP) were assessed at the imaging visit. Smoking, alcohol intake frequency, and physical activity were self-reported at the imaging visit. Hypertension, diabetes, and high cholesterol status at imaging were ascertained by both self-report and linked hospital records (see Supplementary material online, *Table S2*).

### Ascertainment of outcomes

Prevalent conditions existing at the time of imaging were ascertained by both self-report and linked hospital records. Incident diagnoses—occurring for the first time after imaging—were indicated by linked hospital records (see Supplementary material online, *Table S2*). We recorded stroke, myocardial infarction, atrial fibrillation, non-ischaemic cardiomyopathies, heart failure, right-sided valvular disorders (tricuspid or pulmonary), left-sided valvular disorders (mitral or aortic), any CVD, CVD mortality, and all-cause mortality. We also included the following respiratory diseases: asthma, chronic obstructive pulmonary disease (COPD), obstructive sleep apnoea, interstitial lung disease, and bronchiectasis. The study censor date was October 2022, providing an average prospective follow-up time of 4.75 ± 1.52 years. Participants with records of the disease of interest prior to imaging were excluded from modelling for incidence of that outcome.

### Statistical analysis

Analysis was performed using R version 4.2.3 and RStudio Version 2023.03.0 + 386. Relationships were assessed for each ventricular asymmetry type (RV dominant, LV dominant, and mildly LV dominant).

Relationships of asymmetry type with clinical risk factors, such as elevated SBP (>140 mmHg), physical activity, and smoking were characterized using unadjusted logistic regression. Associations between asymmetry and other CMR metrics were assessed using linear regression, with asymmetry entered as a binary exposure variable (no asymmetry set as the reference level) and each CMR metrics inserted in turn as the continuous outcome variable, while adjusting for age, sex, SBP, BMI, deprivation, hypertension, high cholesterol, and diabetes. Associations with prevalent diseases were examined using multivariable logistic regression models with asymmetry category (RV dominant, LV dominant, and mildly LV dominant) set as the outcome, and existing disease as the predictor, adjusted by age, sex, SBP, BMI, and deprivation. Therefore, the odds ratio shows the adjusted odds of having each ventricular asymmetry type given existing cardiorespiratory disease.

Finally, the associations of each asymmetry type with incident cardiovascular events were examined using Cox proportional hazard models, encompassing three layers of adjustment: (i) unadjusted associations between LV and RV dominant ventricular asymmetry and incident cardiovascular events; (ii) demographic and clinical adjustment: age, sex, SBP, BMI, deprivation, hypertension, high cholesterol, and diabetes; and (iii) full adjustment: Model 2 covariates plus additional adjustment for selected CMR metrics previously linked to adverse cardiovascular outcomes—LVEF, RVEF, and LVMi. Results are presented as hazard ratios for incident disease associated with the presence of asymmetry alongside 95% confidence intervals and *P*-values. Multiple testing correction is applied across all significance thresholds using a 5% false discovery rate.

## Results

### Baseline characteristics

The analysis includes 21 545 (48.1%) men and 23 251 (51.9%) women with an overall average age of 64.1 (±7.7) years (*Table 2*). Within the sample, 33.8% had hypertension, 29.3% had high cholesterol, and 5.9% had diabetes. Overall, 2263 (5%) had RV dominant asymmetry, 2658 (6%) had LV dominant asymmetry, and 6656 (15%) had mildly LV dominant asymmetry. Over the follow-up period, 2239 incident CVD events (see Supplementary material online, *Table S3*) and 785 cases of all-cause mortality were observed. Atrial fibrillation was the most commonly observed incident diagnosis with 958 cases, and right-sided valvular disorder was the rarest (81 cases).

**Table 2:** Sample characteristics

Characteristic	Whole sample (*n* = 44 796)	Within normal symmetry (*n* = 39 875)	RV dominant (*n* = 2263)	LV dominant (*n* = 2658)
Age (years)	64.1 (±7.7)	63.9 (±7.7)	65.8 (±7.7)	66.5 (±7.4)
Women	23 251 (51.9%)	20 781 (52.1%)	1185 (52.4%)	1285 (48.3%)
Men	21 545 (48.1%)	19 094 (47.9%)	1078 (47.6%)	1373 (51.7%)
Non-White ethnicity	1336 (3.0%)	1201 (3.0%)	72 (3.2%)	63 (2.4%)
Townsend deprivation index	−2.6 (−3.9, −0.4)	−2.6 (−3.9, −0.4)	−2.5 (−3.8, −0.5)	−2.6 (−3.9, −0.4)
Above median UK deprivation (more deprived)	10 882 (24.3%)	9677 (24.3%)	543 (24.0%)	662 (24.9%)
Body mass index (kg/m^2^)	25.9 (23.5, 28.8)	25.9 (23.5, 28.8)	25.7 (23.3, 28.3)	25.9 (23.4, 28.9)
Obesity (BMI > 30 kg/m^2^)	8028 (17.9%)	7149 (17.9%)	356 (15.7%)	523 (19.7%)
Waist-hip ratio > 1	3039 (6.8%)	2640 (6.6%)	168 (7.4%)	231 (8.7%)
Systolic blood pressure (mmHg)	139.0 (±18.7)	138.7 (±18.5)	136.7 (±17.7)	145.5 (±20.8)
Alcohol intake frequency^a^				
Never	2963 (6.6%)	2583 (6.5%)	161 (7.1%)	219 (8.2%)
Less than once per week	9682 (21.6%)	8631 (21.6%)	506 (22.4%)	545 (20.5%)
Once per week or more	31 821 (71.0%)	28 373 (71.2%)	1574 (69.6%)	1874 (70.5%)
Smoking^a^				
Never smoker	27 780 (62.0%)	24 854 (62.3%)	1432 (63.3%)	1494 (56.2%)
Previous smoker	15 181 (33.9%)	13 400 (33.6%)	748 (33.1%)	1033 (38.9%)
Current smoker	1516 (3.4%)	1344 (3.4%)	61 (2.7%)	111 (4.2%)
Physically active^b^	37 504 (83.7%)	33 489 (84.0%)	1857 (82.1%)	2158 (81.2%)
Ventricular volume ratios				
LV/RV × 100	94.5 (88.3, 101.1)	94.4 (88.9, 100.1)	76.9 (74.3, 78.9)	116.5 (113.5, 122.3)
Hypertension	15 130 (33.8%)	13 150 (33.0%)	741 (32.7%)	1239 (46.6%)
High cholesterol	13 110 (29.3%)	11 381 (28.5%)	693 (30.6%)	1036 (39.0%)
Diabetes	2624 (5.9%)	2274 (5.7%)	132 (5.8%)	218 (8.2%)
Family history of heart disease	24 563 (54.8%)	21 819 (54.7%)	1502 (56.5%)	1242 (54.9%)

There were 6656 (15%) participants with an LV/RV at least 1 SD above the healthy subset mean.

LV, left ventricular end-diastolic volume; RV, right ventricular end-diastolic volume.

^a^Both alcohol intake frequency and smoking had 0.7% missingness.

^b^Physically active is defined as >600 summed metabolic equivalent task minutes per week.

### Ventricular symmetry in the healthy subset

Within the 32 700 healthy participants, the average ventricular ratios (LV/RV) were very similar in men and women and did not exhibit significant age dependency (see Supplementary material online, *Table S1*).

### Associations between ventricular asymmetry and clinical factors

**Figure 1:**
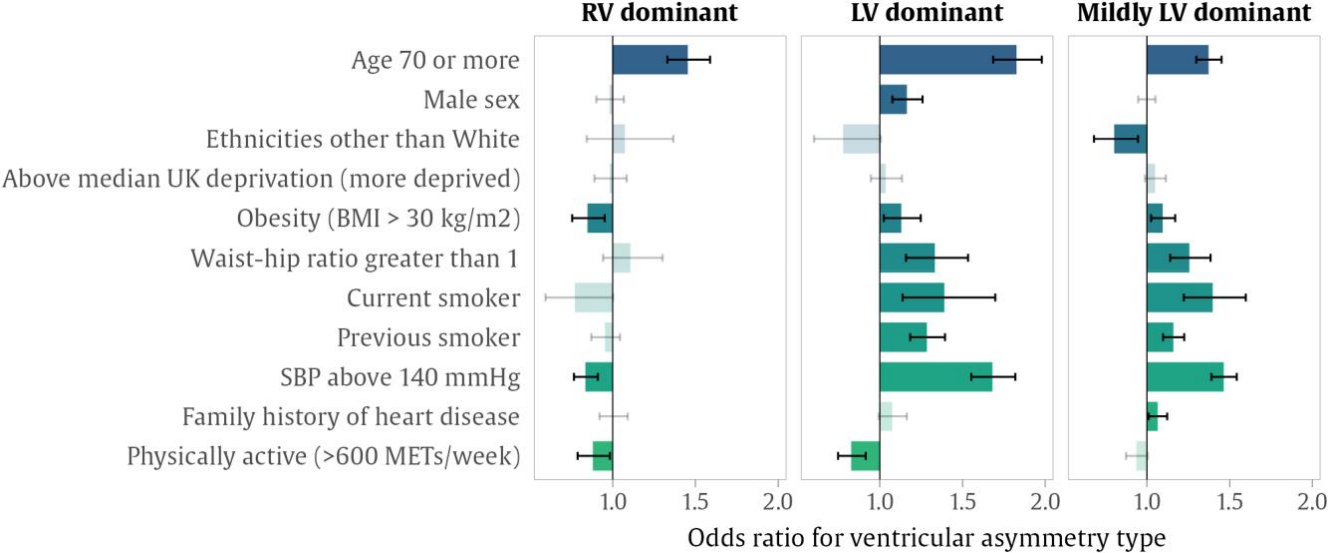
Associations between ventricular asymmetry and risk factors. The bars show the odds ratio for ventricular asymmetry associated with the presence of each risk factor, calculated with logistic regression. The intervals show the 95% confidence interval for the odds ratio, where two-tailed significance was adjusted with a false discovery rate of 5%. Where associations were not significant, these are indicated by lighter shading. BMI, body mass index; LV, left ventricle; MET, metabolic equivalent; RV, right ventricle; SBP, systolic blood pressure.

Participants aged 70 or over were significantly more likely to have LV or RV dominant ventricular asymmetry than younger individuals (*Figure 1* and Supplementary material online, *Table S4*). Left ventricular dominant asymmetry was more frequent in participants with BMI > 30 kg/m^2^, waist-to-hip ratio >1.0, current and previous smoking, and SBP above 140 mmHg. Participants who were physically active (>600 summed metabolic equivalent minutes per week) were less likely to have ventricular asymmetry in either direction.

### Associations between ventricular asymmetry and cardiovascular magnetic resonance metrics

**Figure 2:**
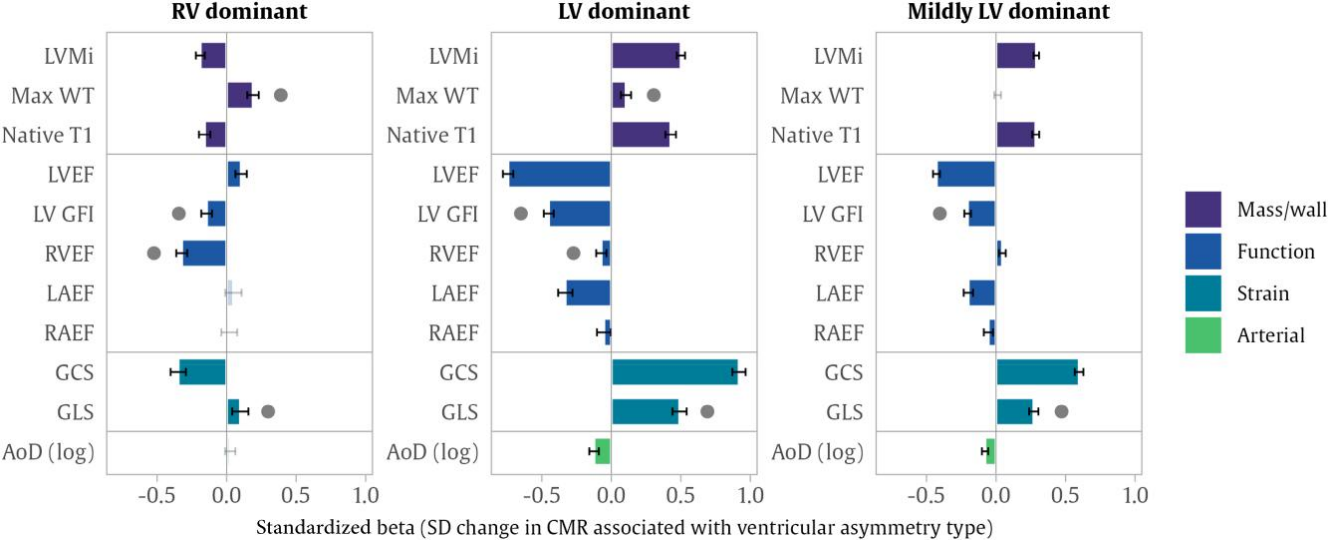
Associations between ventricular asymmetry and other cardiovascular magnetic resonance metrics. The bars show standardized beta coefficients for differences in cardiovascular magnetic resonance measures associated with each type of ventricular asymmetry compared with hearts within the normal symmetry range. Each bar is from a separate linear regression model, relating symmetry category to cardiovascular magnetic resonance metric, adjusted by age, sex, body mass index, systolic blood pressure, smoking, Townsend deprivation index, clinically diagnosed hypertension, diabetes, and high cholesterol. The error bars indicate the 95% confidence interval, with two-tailed significance adjusted with a false discovery rate of 5%. The dots indicate significant cardiovascular magnetic resonance alterations that are found in asymmetry in either direction. AoD, aortic distensibility; GCS, global circumferential strain; GLS, global longitudinal strain; LAEF, left atrial ejection fraction; LVEF, left ventricular ejection fraction; LV GFI, left ventricular global function index; LVMi, left ventricular mass indexed to body surface area; RAEF, right atrial ejection fraction; RVEF, right ventricular ejection fraction; WT, wall thickness.

Left ventricular dominant asymmetry was associated with a pattern of adverse cardiovascular phenotypic alterations (*Figure 2* and Supplementary material online, *Table S5*), characterized by associations with unhealthy myocardial remodelling (higher LVMi, greater max WT, higher myocardial native T1) and worse LV function (lower LVEF, lower LVGFI, deteriorating GLS and GCS). Left ventricular dominant asymmetry was also linked to reduced RV and LA function (lower RVEF and LAEF) and reduced aortic compliance (lower AoD). Notably, these adverse associations also appeared significant (but of smaller magnitude) in the group with mildly LV dominant asymmetry.

Right ventricular dominant asymmetry was associated with significantly lower RVEF, poorer LV function by GLS and LVGFI, and greater max WT. The magnitude of the RVEF effect was much larger in RV dominant than LV dominant asymmetry. Right ventricular dominance was linked to paradoxically higher LVEF and lower myocardial native T1. Mixed effects were observed in the relationships of RV dominant asymmetry with LV strain parameters, with higher (worse) GLS values but a decrease (apparently healthier) in GCS.

### Associations between ventricular asymmetry and prevalent disease

**Figure 3:**
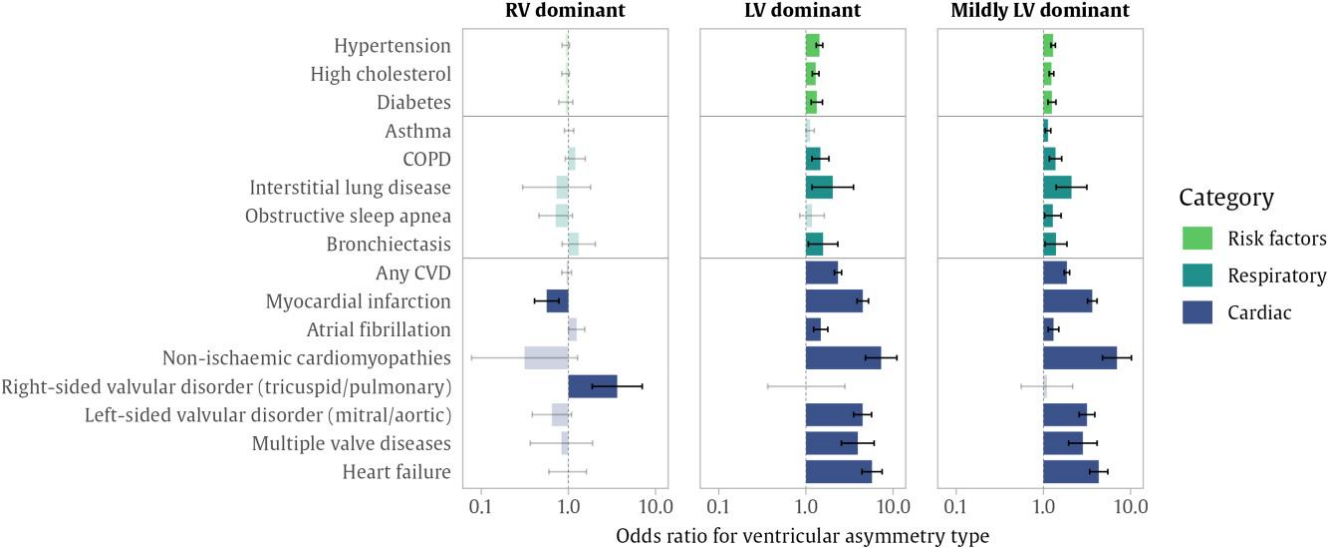
Associations between existing disease and ventricular asymmetry. The bars show the odds ratio for ventricular asymmetry associated with the presence of each existing diagnosis, calculated with logistic regression, and adjusted by age, sex, body mass index, systolic blood pressure, and Townsend deprivation index. The intervals show the 95% confidence interval for the odds ratio, where two-tailed significance was adjusted with a false discovery rate of 5%. Where associations were not significant, these are shown in lighter shade. COPD, chronic obstructive pulmonary disease; CVD, cardiovascular disease.

Left ventricular dominant asymmetry was strongly associated with pre-existing cardiac and respiratory disease, notably myocardial infarction, non-ischaemic cardiomyopathies, left-sided valvular disorders (mitral or aortic), heart failure, interstitial lung diseases, and COPD (*Figure 3* and Supplementary material online, *Table S6*). These associations were also significant in the group with mild LV asymmetry. Participants with RV dominant asymmetry were three times more likely to also have existing right-sided valvular disorders (tricuspid and pulmonary).

### Associations between ventricular asymmetry and incident adverse cardiovascular events

**Figure 4:**
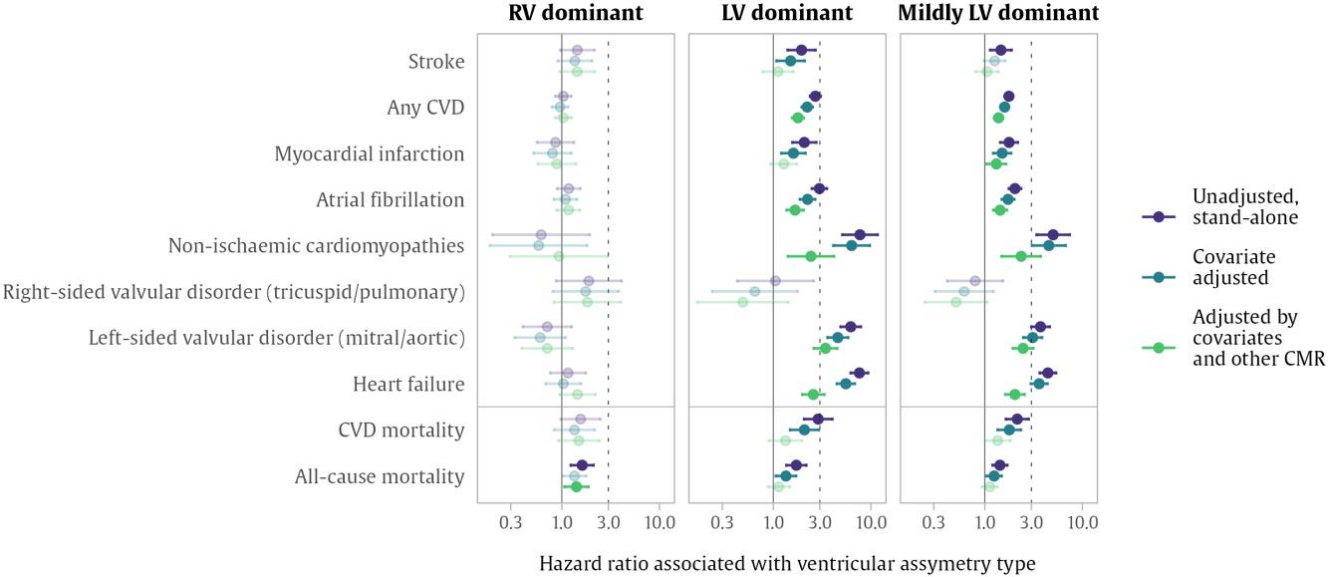
Associations between ventricular asymmetry and incident cardiovascular disease. The dots are hazard ratios for each disease associated with each volume asymmetry type, with 95% confidence interval Cox proportional hazard regression models. The vertical dotted line indicates a three-fold hazard. Model 1 represents unadjusted associations. Model 2 is adjusted by age, sex, systolic blood pressure, body mass index, smoking, Townsend deprivation index, hypertension, high cholesterol, and diabetes. Model 3 is adjusted by Model 2 covariates plus left ventricular mass index, right ventricular ejection fraction, and left ventricular ejection fraction. Associations that are not significant are indicated by faded markers, where the two-tailed significance was adjusted for multiple testing with a false discovery rate of 5%. CMR, cardiovascular magnetic resonance; CVD, cardiovascular disease; LV, left ventricle; RV, right ventricle.

Left ventricular dominant asymmetry as a standalone measurement demonstrated a strong association with increased risk across all outcomes considered except right-sided valvular disorders (*Figure 4* and Supplementary material online, *Table S7*). In fully adjusted models, LV dominance was associated with an increased risk for incident atrial fibrillation and a more than two-fold increased risk for heart failure, non-ischaemic cardiomyopathies, and left-sided valvular disorders. These associations persisted in the group of participants with mild LV dominant asymmetry, with an additional association with myocardial infarction. While RV dominant asymmetry was not associated with any of the incident diseases considered, it was associated with significantly greater risk of all-cause mortality.

## Discussion

### Summary of findings

This study of 44 796 UK Biobank participants characterizes the distribution and clinical associations of ventricular asymmetry and examines the incremental clinical utility of this metric for risk prediction over established clinical and imaging indicators.

In healthy participants, average ventricular symmetry was steady across all ages, while absolute ventricular volumes exhibited significant age dependency. In the overall sample, there was greater ventricular asymmetry (in both directions) with older age.

Left ventricular dominant asymmetry emerged as a reliable indicator for an extensive range of prevalent vascular risk factors, pre-existing cardiorespiratory diseases, and adverse cardiovascular remodelling. Specifically, LV dominant asymmetry demonstrated significant associations with adverse metabolic profile, prevalent ischaemic and non-ischaemic heart diseases, pre-existing obstructive and interstitial lung diseases, and a remodelling pattern indicative of pathologic myocardial alterations (increased max WT, increased LVMi, increased T1), cardiac dysfunction (LV, RV, LA), and poorer aortic compliance (lower AoD).

Importantly, LV dominant asymmetry was associated with significantly increased risk of incident CVDs (atrial fibrillation, non-ischaemic cardiomyopathies, heart failure, left-sided valve diseases) independent of clinical risk factors and conventional CMR measures with established prognostic importance (LVEF, RVEF, LVMi). These relationships were not confined to those with extreme LV asymmetry, and a similar pattern of associations was observed in individuals with mildly LV dominant asymmetry. Right ventricular dominant asymmetry was associated with ageing, reduced RV function, prevalent right-sided valve disorders, and all-cause mortality.

### Comparison with existing literature

Ventricular volume ratio, as a measure of ventricular asymmetry, represents a simple and readily calculable imaging biomarker derived from existing ventricular measurements. Prior literature proposes that RV dominant asymmetry, assessed by either volume or diameter ratio plays a significant role in the risk stratification of specific conditions, such as pulmonary embolism and pulmonary hypertension.^14–17^ Our study provides new insights by examining associations of both LV and RV dominant asymmetry and by considering relationships in a population sample without selection on any disease. We corroborate previously reported associations of RV dominant asymmetry with all-cause death. We additionally demonstrate the broader utility of LV dominant asymmetry in the general population where left-sided heart diseases present a greater burden. Our findings extend the remit of ventricular asymmetry beyond the limited conditions proposed in previous research and highlight the incremental prognostic value of LV dominant asymmetry in the general population over established clinical and imaging indicators.

Our study illustrates the adverse cardiovascular remodelling pattern associated with LV dominant asymmetry, independent of clinical risk factors. These findings indicate that LV dominant asymmetry presents a reflection of a range of maladaptive, potentially subclinical, cardiac alterations. The pathway driving these relationships is multifaceted. Chronic pressure overload in the systemic circulation could be a potential driver, instigating LV hypertrophy and consequential tissue level modifications.^18^ Left ventricular dominant asymmetry and its association with non-ischaemic cardiomyopathies may suggest key anatomical and gene expression differences of the diseases within this category (e.g. dilated cardiomyopathy).^19^ Remodelling pattern differences between the LV and RV can be attributed, at least in part to their embryological origin.^20^ The LV arises from the first heart field, and the RV originates from the second heart field. Genetic predispositions, variations in proteomic profiles, and differing pressure conditions in the ventricles within the circulatory system may all contribute to the asymmetrical involvement observed in a large proportion of patients.^21^ Our study noted an association of LV dominant asymmetry with elevated T1 values, a surrogate marker for diffuse myocardial fibrosis.^22^ Fibrosis of the myocardium can cause an increase in ventricular stiffness, subsequently leading to systolic and diastolic dysfunction and ultimately increased risk of heart failure and cardiac-related mortality.

Moving beyond cardiac remodelling patterns, we further elucidated associations between LV dominant asymmetry and prevalent cardiometabolic risk factors, including older age and higher BMI. Both ageing and obesity are well-documented risk factors for CVD, with their impact manifesting through a series of pathophysiological changes, including hypertension, diabetes, and dyslipidaemia, together or independently.^23^ Given that these conditions primarily impact the systemic circulation, the resultant cardiovascular remodelling will preferentially impact the LV and may lead to LV dominant ventricular asymmetry.

Moreover, our study provides compelling evidence that LV dominant asymmetry is significantly associated with both prevalent and future cardiorespiratory disease, even after adjusting for conventional imaging-derived biomarkers, such as LVEF or LVMi. This observation adds a prognostic dimension to our understanding of ventricular asymmetry, underscoring its potential value as a risk stratification tool. This also suggests that the LV dominant asymmetry provides additional prognostic information beyond traditional measures of cardiac function. As an example, in heart failure the association with LV dominance appears to be multifactorial with one of the leading factors being the activation of the renin–angiotensin–aldosterone system which provokes significant LV remodelling.^24^ Left ventricular dominant asymmetry was strongly associated with the development of AF with that being elucidated by LV dominance inducing left atrial remodelling driven by atrial fibrosis.^25,26^ The molecular and cellular mechanisms that connect ventricular asymmetry to cardiac remodelling and disease development warrant further investigation.

Our study found a significant association between RV dominant remodelling and an increased risk of all-cause mortality. This association was seen in the absence of an associated increase in most prevalent or incident CVD. While an association with prevalent right-sided valvular disease was observed, its rarity makes it unlikely to be the primary explanation for the observed increased mortality. Instead, the observed RV remodelling may be a more global indicator of systemic or non-cardiac conditions that contribute to increased all-cause mortality. Indeed, adverse RV remodelling has been previously associated with systemic conditions, including liver disease,^27,28^ renal disease,^29^ and cancer,^30^ indicating a potential multisystem involvement that could influence overall patient health and mortality risk. Furthermore, biological processes like inflammation, oxidative stress, or fibrosis, all of which have been linked with RV remodelling,^31^ could also be contributing to this increased mortality. This supports the idea that RV remodelling could be a surrogate marker of these underlying processes, which have systemic implications beyond the cardiovascular system.

### Clinical implications

Ventricular asymmetry may serve as an important additional metric in patient assessment, enhancing existing models of risk stratification. The observed associations between ventricular asymmetry, specifically LV dominant asymmetry, and adverse cardiovascular outcomes suggest its potential as a marker for early or subclinical disease. This utility is further emphasized by its prognostic value independent of established imaging biomarkers, such as LVEF and LVMi. Given the simplicity of deriving this metric, its integration into clinical routine would not require significant additional resource investment and may lead to substantial long-term savings, which merits formal evaluation in dedicated cost-effectiveness studies.

### Study limitations

This analysis presents associations of ventricular asymmetry in a low-risk cohort. Further validation of the observed relationships in clinical cohorts with greater pathology burden is required. The UK Biobank sample has limited ethnic diversity, and our analysis cannot comment on any variations in relationships across ethnic groups. Furthermore, our sample is limited to a middle-aged adult cohort, and there may be variations in the reported relationships in the very young or very old that are not evaluated in this analysis. Finally, ventricular asymmetry may provide valuable prognostic information, and its interpretation should always be considered alongside other clinical and imaging markers to avoid misclassification in patients with globally enlarged hearts.

## Conclusions

The observations from this large-scale imaging study enrich our understanding of cardiovascular remodelling and disease progression. Our study underscores the significant association between ventricular asymmetry, specifically LV dominant asymmetry, and prevalent and incident cardiorespiratory diseases, and its prognostic value beyond traditional risk factors and established CMR metrics.

## Supplementary Material

oeae059_Supplementary_Data

## Data Availability

This research was conducted using the UK Biobank resource under access application 2964. The UK Biobank will make the data available to all bona fide researchers for all types of health-related research that is in the public interest, without preferential or exclusive access for any persons. All researchers will be subject to the same application process and approval criteria as specified by the UK Biobank. For more details on the access procedure, see the UK Biobank website: http://www.ukbiobank.ac.uk/register-apply.

## Lead author biography

Celeste McCracken is a statistician, data scientist, and researcher at the University of Oxford, affiliated with the Oxford Centre for Clinical Magnetic Resonance Research, the Department of Cardiovascular Medicine, and the Nuffield Department of Population Health. Celeste McCracken has lectured in statistics, data analysis, and programming and worked in healthcare consulting and data coaching. In her work spanning cardiovascular health, multi-organ health, and multi-morbidity, she specializes in integrating large, complex medical data sets to support evidence-based decision-making. Her work focuses on using data to enhance health services delivery, remove inequalities, and improve the longer-term trajectory of health outcomes.
