# Supplementary material for: Ventricular volume asymmetry as a novel imaging biomarker for disease discrimination and outcome prediction
Source: Eur Heart J Open. 2024 Jul 25;4(4):oeae059. doi: 10.1093/ehjopen/oeae059 (PMC11306927; doi:10.1093/ehjopen/oeae059)
Supplement: oeae059_Supplementary_Data [file oeae059_supplementary_data.pdf]

Supplementary Materials

Supplementary Figure 1: Study sample

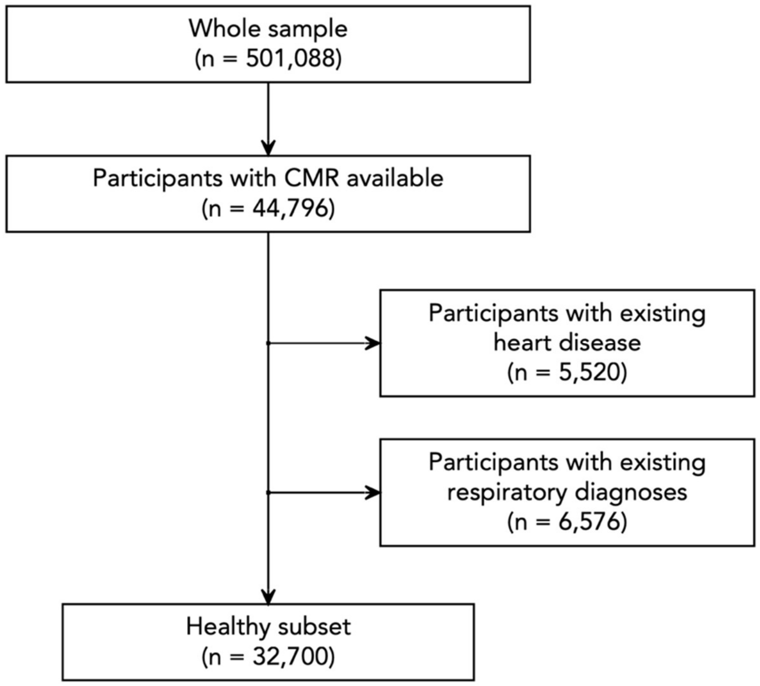

CMR = cardiovascular magnetic resonance.

(**back to Methods**)

Supplementary Table 1: Ventricular ratio percentiles by sex and age

Sex	Age group	LV/RV x 100
Female	Less than 50	96.3 [83.5, 110.6]
(n= 17,687)	50-54	96.2 [82.1, 111.3]
54.1%	55-59	96.3 [82.8, 111.6]
	60-64	96.3 [81.8, 112.4]
	65-69	95.9 [80.9, 113.9]
	70-74	95.6 [80.6, 115.2]
	75 or more	95.5 [80.1, 115.9]
Male	Less than 50	92.0 [78.5, 105.8]
(n= 15, 013)	50-54	91.7 [79.5, 105.8]
45.9%	55-59	91.9 [79.2, 106.4]
	60-64	92.1 [78.7, 109.0]
	65-69	92.4 [78.6, 108.8]
	70-74	92.6 [78.7, 111.1]
	75 or more	91.6 [76.3, 112.8]

Supplementary Table 1 footnote: Entries are median ventricular asymmetry in the healthy subset, by sex and age [5th percentile, 95th percentile]. RV = right ventricular end-diastolic volume, LV = left ventricular end-diastolic volume. Ratios can be calculated with either raw or indexed ventricular volumes.

(**back to Methods**, **back to Results**)

Supplementary Table 2: Disease definitions

(back to Methods)

Disease	Source	Code / UK Biobank Field ID
Stroke	Algorithm	42006
	Diagnosed by doctor (6150, 6152)	3, 4056
	First occurrences	131360, 131362, 131366, 131368
	ICD10	I60, I61, I63, I64
	ICD9	430, 431, 434, 436
	Self-report (20002)	1081, 1086, 1491, 1583
Myocardial infarction	Algorithm	42000
	Diagnosed by doctor (6150, 6152)	1, 3894
	First occurrences	131298, 131300, 131302
	ICD10	I21, I22, I23, I241, I252
	ICD9	410, 411, 412, 429
	Self-report (20002)	1075
Atrial fibrillation	First occurrences	131350
	ICD10	I48, I480, I481, I482, I489
	ICD9	4273
	Self-report (20002)	1471
Non-ischemic cardiomyopathies	First occurrences	131288, 131292, 131338, 131340
	ICD10	I11, I13, I42, I43
	ICD9	4020, 4021, 4029, 4040, 4041, 4049, 4250, 4253, 4255
	Self-report (20002)	1079, 1588
Heart failure	First occurrences	131354
	ICD10	I500, I501, I509
	ICD9	4280, 4281, 4289
	Self-report (20002)	1076
Left-sided valvular	First occurrences	131276, 131278, 131322, 131324
	ICD10	I05, I06, I34, I35
Right-sided valvular	First occurrences	131280, 131326, 131328
	ICD10	I07, I36, I37
Diabetes	Diagnosed by doctor	2443, 2976, 2976, 4041, 2986
	First occurrences	130706, 130708, 130712, 130714, 130706, 130708
	ICD10	E10, E11, E13, E14, G590, G632, H280, H360, M142, N083, O240, O241, O243, O244, O249, Y423, E10, O240, E11, O241, O244
	ICD9	6480
	Medications (20003)	1140883066
	Medications (6153, 6177)	3, 3
	Self-report (20002)	1220, 1222, 1223, 1222, 1223
Asthma	Diagnosed by doctor	22147, 3786
	First occurrences	131494, 131496
	ICD10	J45, J46
	Self-report (20002)	1111
COPD	Diagnosed by doctor	22130, 22150
	First occurrences	131486, 131488, 131490, 131492
	ICD10	J41, J42, J43, J44
	Self-report (20002)	1112, 1113
Obstructive sleep apnoea	ICD10	E662, G473
	Self-report (20002)	1123
Interstitial lung disease	First occurrences	131522, 131524, 131526, 131528
	ICD10	J80, J81, J82, J84
	Self-report (20002)	1115
	ICD10	E662, G473
	Self-report (20002)	1123

Bronchiectasis	First occurrences	131498
	ICD10	J47
	Self-report (20002)	1114
Hypertension	Diagnosed by doctor (6150, 6152)	2966, 4
	First occurrences	131286
	ICD10	I10
	Medications (6153, 6177)	2
	Self-report (20002)	1065, 1072
High cholesterol	First occurrences	130814
	ICD10	E780, E782, E783, E784, E785
	Medications (6153, 6177)	1
	Self-report (20002)	1473

Supplementary Table 2 footnote: ICD10 codes are drawn from fields 41270, 41280, 41234 and 41259; ICD9 codes are drawn from 41271 and 41281, Where a 3-digit code is given, this includes all 4-digit sub-codes, for example, E10 includes E100, E101 and E102.

Supplementary Table 3: Prevalent and Incident disease counts

Condition	Whole sample (n= 44,796)	Within normal symmetry (n= 39,875)	RV dominant (n= 2,263)	LV dominant (n= 2,658)
**Existing/prevalent at imaging**				
Stroke	914 (2.0%)	778 (2.0%)	49 (2.2%)	87 (3.3%)
Asthma	6,232 (13.9%)	5,532 (13.9%)	312 (13.8%)	388 (14.6%)
COPD	949 (2.1%)	798 (2.0%)	62 (2.7%)	89 (3.3%)
Interstitial lung disease	119 (0.3%)	99 (0.2%)	5 (0.2%)	15 (0.6%)
Obstructive sleep apnoea	569 (1.3%)	507 (1.3%)	21 (0.9%)	41 (1.5%)
Bronchiectasis	287 (0.6%)	236 (0.6%)	22 (1.0%)	29 (1.1%)
Ischemic heart disease	2,797 (6.2%)	2,245 (5.6%)	141 (6.2%)	411 (15.5%)
Myocardial infarction	1,148 (2.6%)	855 (2.1%)	40 (1.8%)	253 (9.5%)
Atrial fibrillation	1,358 (3.0%)	1,129 (2.8%)	97 (4.3%)	132 (5.0%)
Non-ischemic cardiomyopathies	108 (0.2%)	72 (0.2%)	2 (0.1%)	34 (1.3%)
Right-sided valvular disorder (tricuspid/pulmonary)	61 (0.1%)	46 (0.1%)	11 (0.5%)	4 (0.2%)
Left-sided valvular disorder (mitral/aortic)	401 (0.9%)	290 (0.7%)	15 (0.7%)	96 (3.6%)
Multiple valve diseases	124 (0.3%)	91 (0.2%)	6 (0.3%)	27 (1.0%)
Heart failure	285 (0.6%)	186 (0.5%)	17 (0.8%)	82 (3.1%)
**Incident/ after imaging**				
Stroke	371 (0.8%)	306 (0.8%)	25 (1.1%)	40 (1.5%)
Any CVD	2,239 (5.0%)	1,856 (4.7%)	110 (4.9%)	273 (10.3%)
Myocardial infarction	502 (1.1%)	427 (1.1%)	21 (0.9%)	54 (2.0%)
Atrial fibrillation	958 (2.1%)	759 (1.9%)	53 (2.3%)	146 (5.5%)
Non-ischemic cardiomyopathies	102 (0.2%)	66 (0.2%)	3 (0.1%)	33 (1.2%)
Right-sided valvular disorder (tricuspid/pulmonary)	81 (0.2%)	69 (0.2%)	7 (0.3%)	5 (0.2%)
Left-sided valvular disorder (mitral/aortic)	343 (0.8%)	238 (0.6%)	12 (0.5%)	93 (3.5%)
Heart failure	421 (0.9%)	266 (0.7%)	23 (1.0%)	132 (5.0%)
CVD mortality	157 (0.4%)	121 (0.3%)	26 (1.0%)	10 (0.4%)
All-cause mortality	785 (1.8%)	650 (1.6%)	58 (2.6%)	77 (2.9%)
Follow-up time (years)	4.75 (± 1.5)			

COPD = chronic obstructive pulmonary disease, CVD = cardiovascular disease

(**Back to Results**)

Supplementary Table 4: Ventricular asymmetry by clinical factors

Feature	RV dominant	LV dominant	Mildly LV dominant
Age 70 or more	1.45* [1.33, 1.59] 1.17×10^{-16}	1.83* [1.69, 1.98] 6.28×10^{-49}	1.37* [1.30, 1.45] 5.98×10^{-29}
Male sex	0.98 [0.90, 1.07] 0.6531	1.16* [1.08, 1.26] 1.54×10^{-4}	1.00 [0.95, 1.05] 0.9310
Ethnicities other than White	1.07 [0.84, 1.37] 0.5660	0.78 [0.60, 1.01] 0.0551	0.80* [0.68, 0.95] 0.0088
Above median UK deprivation (more deprived)	0.98 [0.89, 1.08] 0.7243	1.04 [0.95, 1.13] 0.4515	1.05 [0.99, 1.11] 0.1174
Obesity (BMI > 30 kg/m2)	0.85* [0.75, 0.95] 0.0052	1.13* [1.02, 1.25] 0.0153	1.10* [1.02, 1.17] 0.0073
Waist-hip ratio greater than 1	1.11 [0.94, 1.30] 0.2170	1.33* [1.16, 1.53] 5.99×10^{-5}	1.26* [1.14, 1.38] 3.88×10^{-6}
Current smoker	0.77 [0.59, 1.00] 0.0518	1.39* [1.14, 1.70] 0.0013	1.40* [1.22, 1.60] 9.26×10^{-7}
Previous smoker	0.95 [0.87, 1.04] 0.3043	1.28* [1.18, 1.39] 2.05×10^{-9}	1.16* [1.10, 1.23] 1.32×10^{-7}
SBP above 140 mmHg	0.83* [0.77, 0.91] 4.10×10^{-5}	1.68* [1.55, 1.82] 7.26×10^{-38}	1.46* [1.39, 1.54] 2.03×10^{-46}
Family history of heart disease	1.00 [0.92, 1.09] 0.9610	1.07 [0.99, 1.16] 0.0735	1.07* [1.01, 1.12] 0.0184
Physically active (>600 METs/week)	0.88* [0.79, 0.98] 0.0238	0.83* [0.75, 0.91] 2.28×10^{-4}	0.94 [0.87, 1.00] 0.0654

Supplementary Table 4 footnote: Entries are odds ratios for ventricular asymmetry associated with each clinical feature, along with 95% confidence intervals, and p-values from unadjusted logistic regression models. An asterisk (*) indicates where p-values are significant after multiple testing adjustment with a 5% false discovery rate.

(**back to Results**)

Supplementary Table 5: Associations between ventricular asymmetry and other CMR metrics

CMR Metric	RV dominant	LV dominant	Mildly LV dominant
LVMi	-0.19* [-0.22, -0.16] 1.00×10^{-30}	0.50* [0.47, 0.53] 2.15×10^{-241}	0.29* [0.27, 0.31] 2.61×10^{-182}
Max WT	0.19* [0.15, 0.23] 2.69×10^{-19}	0.11* [0.07, 0.14] 1.75×10^{-8}	0.01 [-0.01, 0.04] 0.3534
Native T1	-0.16* [-0.20, -0.12] 4.10×10^{-14}	0.43* [0.39, 0.46] 5.78×10^{-108}	0.29* [0.26, 0.31] 7.56×10^{-110}
LVEF	0.10* [0.06, 0.14] 5.98×10^{-7}	-0.74* [-0.78, -0.71] $<1.00 \times 10^{-200}$	-0.43* [-0.45, -0.40] 7.33×10^{-249}
LV GFI	-0.14* [-0.18, -0.11] 2.63x10-13	-0.45* [-0.49, -0.41] 1.70×10^{-135}	-0.20* [-0.23, -0.18] 8.90×10^{-64}
RVEF	-0.32* [-0.36, -0.28] 6.65×10^{-58}	-0.07* [-0.11, -0.04] 1.17×10^{-4}	0.05* [0.02, 0.07] 2.84×10^{-4}
LAEF	0.05 [-0.01, 0.11] 0.1039	-0.33* [-0.38, -0.28] 1.72×10^{-36}	-0.20* [-0.23, -0.17] 3.57×10^{-31}
RAEF	0.02 [-0.04, 0.08] 0.5127	-0.05* [-0.10, -0.00] 0.0377	-0.05* [-0.09, -0.02] 0.001
GCS	-0.35* [-0.40, -0.29] 2.76×10^{-35}	0.92* [0.87, 0.97] $<1.00 \times 10^{-200}$	0.60* [0.57, 0.63] $<1.00 \times 10^{-200}$
GLS	0.10* [0.04, 0.16] 9.47×10^{-4}	0.49* [0.44, 0.54] 3.54×10^{-80}	0.27* [0.24, 0.30] 1.20×10^{-57}
AoD (log)	0.03 [-0.01, 0.06] 0.1677	-0.12* [-0.16, -0.09] 1.57×10^{-12}	-0.08* [-0.10, -0.06] 1.21×10^{-11}

Supplementary Table 5 footnote: Entries are standardised beta coefficients with 95% confidence intervals and p-values for differences in CMR measures associated with each type of ventricular asymmetry compared to hearts within the normal symmetry range. Each cell is from a separate linear regression model, relating symmetry category to CMR metric, adjusted by age, sex, body mass index, systolic blood pressure, smoking, Townsend deprivation index, clinically diagnosed hypertension, diabetes and high cholesterol. An asterisk (*) indicates p-values that are significant after multiple testing adjustment with a false discovery rate of 5%. (**back to Results**)

Supplementary Table 6: Associations between ventricular asymmetry and existing disease

	RV dominant		LV dominant		Mildly LV dominant	
**Condition**	**Model 1**	**Model 2**	**Model 1**	**Model 2**	**Model 1**	**Model 2**
Hypertension	0.95<br>[0.87, 1.04]<br>0.2872	0.93<br>[0.85, 1.03]<br>0.1687	1.78*<br>[1.64, 1.92]<br>3.51×10^{-46}	1.43*<br>[1.31, 1.56]<br>3.30×10^{-16}	1.47*<br>[1.39, 1.55]<br>3.85×10^{-45}	1.29*<br>[1.22, 1.37]<br>5.75×10^{-18}
High cholesterol	1.07<br>[0.98, 1.17]<br>0.1454	0.93<br>[0.84, 1.03]<br>0.1436	1.59*<br>[1.47, 1.72]<br>2.06×10^{-29}	1.29*<br>[1.19, 1.41]<br>9.18×10^{-9}	1.35*<br>[1.28, 1.42]<br>2.88×10^{-26}	1.24*<br>[1.17, 1.31]<br>3.65×10^{-12}
Diabetes	1.00<br>[0.83, 1.19]<br>0.9590	0.93<br>[0.77, 1.12]<br>0.4603	1.48*<br>[1.28, 1.70]<br>1.33×10^{-7}	1.33*<br>[1.15, 1.55]<br>1.54×10^{-4}	1.30*<br>[1.17, 1.44]<br>6.64×10^{-7}	1.25*<br>[1.13, 1.39]<br>3.01×10^{-5}
Asthma	0.99<br>[0.87, 1.12]<br>0.8601	1.02<br>[0.90, 1.15]<br>0.7725	1.06<br>[0.95, 1.19]<br>0.2924	1.12<br>[1.00, 1.25]<br>0.0522	1.10*<br>[1.02, 1.18]<br>0.0140	1.13*<br>[1.05, 1.22]<br>0.0013
COPD	1.32<br>[1.02, 1.72]<br>0.0358	1.20<br>[0.92, 1.56]<br>0.1761	1.66*<br>[1.33, 2.08]<br>7.04×10^{-6}	1.47*<br>[1.17, 1.84]<br>8.10×10^{-4}	1.46*<br>[1.24, 1.71]<br>4.52×10^{-6}	1.38*<br>[1.17, 1.62]<br>1.17×10^{-4}
Interstitial lung disease	0.82<br>[0.34, 2.02]<br>0.6721	0.73<br>[0.30, 1.80]<br>0.4949	2.29*<br>[1.33, 3.95]<br>0.0027	2.03*<br>[1.18, 3.52]<br>0.0111	2.20*<br>[1.47, 3.30]<br>1.17×10^{-4}	2.10*<br>[1.40, 3.15]<br>3.28×10^{-4}
Obstructive sleep apnea	0.72<br>[0.46, 1.11]<br>0.1375	0.71<br>[0.46, 1.11]<br>0.1351	1.23<br>[0.90, 1.70]<br>0.1970	1.18<br>[0.85, 1.63]<br>0.3303	1.27*<br>[1.02, 1.58]<br>0.0290	1.28*<br>[1.03, 1.59]<br>0.0257
Bronchiectasis	1.57<br>[1.01, 2.42]<br>0.0443	1.31<br>[0.85, 2.04]<br>0.2249	1.79*<br>[1.22, 2.63]<br>0.0031	1.57*<br>[1.07, 2.32]<br>0.0223	1.52*<br>[1.14, 2.02]<br>0.0041	1.39*<br>[1.04, 1.86]<br>0.0249
Any CVD	1.08<br>[0.96, 1.23]<br>0.2091	0.95<br>[0.84, 1.08]<br>0.4653	2.58*<br>[2.35, 2.83]<br>1.78×10^{-89}	2.33*<br>[2.12, 2.57]<br>3.62×10^{-67}	1.91*<br>[1.79, 2.05]<br>2.38×10^{-76}	1.86*<br>[1.73, 2.00]<br>5.29×10^{-66}
Myocardial infarction	0.67*<br>[0.49, 0.92]<br>0.0147	0.56*<br>[0.41, 0.78]<br>4.62×10^{-4}	4.85*<br>[4.19, 5.61]<br>2.38×10^{-100}	4.49*<br>[3.86, 5.23]<br>2.40×10^{-84}	3.57*<br>[3.16, 4.04]<br>4.48×10^{-92}	3.64*<br>[3.20, 4.13]<br>2.88×10^{-89}
Atrial fibrillation	1.47*<br>[1.19, 1.81]<br>3.83x10-4	1.24<br>[1.00, 1.54]<br>0.0468	1.74*<br>[1.45, 2.10]<br>3.15×10^{-9}	1.48*<br>[1.23, 1.79]<br>4.16×10^{-5}	1.38*<br>[1.20, 1.58]<br>4.88×10^{-6}	1.31*<br>[1.14, 1.50]<br>1.82×10^{-4}
Non-ischemic cardiomyopathies	0.35<br>[0.09, 1.44]<br>0.1459	0.31<br>[0.08, 1.27]<br>0.1047	7.37*<br>[4.90, 11.08]<br>8.59×10^{-22}	7.31*<br>[4.83, 11.07]<br>6.02×10^{-21}	6.70*<br>[4.58, 9.78]<br>8.33×10^{-23}	6.98*<br>[4.76, 10.24]<br>2.32×10^{-23}
Right-sided valvular disorder (tricuspid/pulmonary)	4.15*<br>[2.16, 7.98]<br>2.00×10^{-5}	3.63*<br>[1.88, 7.03]<br>1.29×10^{-4}	1.11<br>[0.40, 3.07]<br>0.8366	1.01<br>[0.36, 2.80]<br>0.9833	1.12<br>[0.57, 2.21]<br>0.7360	1.10<br>[0.56, 2.17]<br>0.7898
Left-sided valvular disorder (mitral/aortic)	0.73<br>[0.43, 1.22]<br>0.2304	0.65<br>[0.38, 1.09]<br>0.0990	5.14*<br>[4.07, 6.49]<br>3.33×10^{-43}	4.48*<br>[3.53, 5.67]<br>2.17×10^{-35}	3.37*<br>[2.74, 4.13]<br>3.18×10^{-31}	3.16*<br>[2.57, 3.89]<br>1.08×10^{-27}
Multiple valve diseases	0.96<br>[0.42, 2.17]<br>0.9136	0.83<br>[0.36, 1.89]<br>0.6580	4.45*<br>[2.90, 6.83]<br>8.51×10^{-12}	3.94*<br>[2.55, 6.07]<br>5.66×10^{-10}	2.95*<br>[2.03, 4.28]<br>1.33×10^{-8}	2.84*<br>[1.95, 4.13]<br>5.35×10^{-8}
Heart failure	1.19<br>[0.73, 1.95]<br>0.4807	0.98<br>[0.60, 1.61]<br>0.9347	6.58*<br>[5.07, 8.52]<br>6.54×10^{-46}	5.74*<br>[4.40, 7.49]<br>7.39×10^{-38}	4.41*<br>[3.49, 5.59]<br>5.69×10^{-35}	4.32*<br>[3.40, 5.49]<br>5.91×10^{-33}

Supplementary Table 6 footnote: Entries are odds ratio for ventricular asymmetry associated with the presence of each existing diagnosis, along with 95% confidence intervals and p-values, calculated with logistic regression. Model 1 is the crude, unadjusted association. Model 2 is adjusted by age, sex, body mass index, systolic blood pressure and Townsend deprivation index. An asterisk (*) indicates p-values that are significant after multiple testing adjustment with a false discovery rate of 5%. (**back to Results**)

Supplementary Table 7: Associations between ventricular ratio and incident disease

	RV dominates			LV dominates			Mildly LV dominant		
	**Model 1**	**Model 2**	**Model 3**	**Model 1**	**Model 2**	**Model 3**	**Model 1**	**Model 2**	**Model 3**
Stroke	1.44<br>[0.96, 2.16]<br>0.0773	1.36<br>[0.91, 2.05]<br>0.1378	1.44<br>[0.95, 2.17]<br>0.0850	1.95*<br>[1.40, 2.70]<br>$6.97\text{x}10^{-5}$	1.50*<br>[1.08, 2.09]<br>0.0159	1.12<br>[0.77, 1.62]<br>0.5492	1.46*<br>[1.14, 1.88]<br>0.0031	1.26<br>[0.98, 1.63]<br>0.0731	1.05<br>[0.80, 1.39]<br>0.7084
Any CVD	1.04<br>[0.85, 1.25]<br>0.7219	0.96<br>[0.79, 1.16]<br>0.6675	1.04<br>[0.85, 1.26]<br>0.7153	2.70*<br>[2.38, 3.06]<br>$2.91\text{x}10^{-53}$	2.23*<br>[1.96, 2.53]<br>$3.86\text{x}10^{-34}$	1.79*<br>[1.56, 2.06]<br>$1.59\text{x}10^{-16}$	1.77*<br>[1.60, 1.95]<br>$6.72\text{x}10^{-29}$	1.60*<br>[1.44, 1.77]<br>$1.38\text{x}10^{-19}$	1.38*<br>[1.24, 1.54]<br>$2.75\text{x}10^{-9}$
Myocardial infarction	0.86<br>[0.56, 1.33]<br>0.5037	0.80<br>[0.51, 1.26]<br>0.3373	0.89<br>[0.57, 1.40]<br>0.6094	2.08*<br>[1.57, 2.76]<br>$3.81\text{x}10^{-7}$	1.61*<br>[1.21, 2.14]<br>0.0011	1.28<br>[0.94, 1.75]<br>0.1163	1.77*<br>[1.44, 2.18]<br>$6.44\text{x}10^{-8}$	1.51*<br>[1.22, 1.86]<br>$1.46\text{x}10^{-4}$	1.31*<br>[1.05, 1.65]<br>0.0181
Atrial fibrillation	1.18<br>[0.89, 1.55]<br>0.2499	1.09<br>[0.83, 1.44]<br>0.5440	1.17<br>[0.88, 1.55]<br>0.2748	2.97*<br>[2.49, 3.54]<br>$9.63\text{x}10^{-34}$	2.24*<br>[1.87, 2.68]<br>$1.24\text{x}10^{-18}$	1.67*<br>[1.36, 2.05]<br>$7.01\text{x}10^{-7}$	2.04*<br>[1.76, 2.35]<br>$3.67\text{x}10^{-22}$	1.73*<br>[1.49, 2.00]<br>$2.66\text{x}10^{-13}$	1.43*<br>[1.22, 1.68]<br>$9.69\text{x}10^{-6}$
Non-ischemic cardiomyopathies	0.61<br>[0.19, 1.94]<br>0.4058	0.58<br>[0.18, 1.83]<br>0.3508	0.93<br>[0.29, 2.98]<br>0.9089	7.73*<br>[5.10, 11.70]<br>$4.39\text{x}10^{-22}$	6.34*<br>[4.13, 9.74]<br>$2.95\text{x}10^{-17}$	2.43*<br>[1.41, 4.19]<br>0.0014	5.02*<br>[3.40, 7.40]<br>$4.31\text{x}10^{-16}$	4.53*<br>[3.05, 6.74]<br>$7.98\text{x}10^{-14}$	2.36*<br>[1.49, 3.74]<br>$2.62\text{x}10^{-4}$
Right-sided valvular disorder (tricuspid/pulmonary)	1.89<br>[0.87, 4.11]<br>0.1071	1.75<br>[0.81, 3.82]<br>0.1571	1.82<br>[0.83, 4.02]<br>0.1363	1.05<br>[0.42, 2.60]<br>0.9157	0.65<br>[0.24, 1.78]<br>0.3994	0.49<br>[0.17, 1.43]<br>0.1902	0.80<br>[0.41, 1.54]<br>0.4980	0.61<br>[0.31, 1.23]<br>0.1716	0.51<br>[0.24, 1.06]<br>0.0724
Left-sided valvular disorder (mitral/aortic)	0.71<br>[0.40, 1.26]<br>0.2423	0.60<br>[0.33, 1.09]<br>0.0957	0.71<br>[0.39, 1.30]<br>0.2657	6.22*<br>[4.90, 7.89]<br>$3.66\text{x}10^{-51}$	4.58*<br>[3.60, 5.84]<br>$1.08\text{x}10^{-34}$	3.43*<br>[2.59, 4.52]<br>$4.15\text{x}10^{-18}$	3.72*<br>[3.00, 4.62]<br>$1.61\text{x}10^{-32}$	3.09*<br>[2.48, 3.86]<br>$1.06\text{x}10^{-23}$	2.47*<br>[1.94, 3.14]<br>$2.08\text{x}10^{-13}$
Heart failure	1.16<br>[0.76, 1.76]<br>0.4968	1.04<br>[0.68, 1.58]<br>0.8713	1.45<br>[0.95, 2.22]<br>0.0883	7.63*<br>[6.21, 9.37]<br>$2.37\text{x}10^{-83}$	5.53*<br>[4.48, 6.83]<br>$7.46\text{x}10^{-57}$	2.57*<br>[1.98, 3.34]<br>$1.46\text{x}10^{-12}$	4.43*<br>[3.65, 5.37]<br>$9.69\text{x}10^{-52}$	3.61*<br>[2.96, 4.39]<br>$1.70\text{x}10^{-37}$	2.04*<br>[1.62, 2.55]<br>$8.69\text{x}10^{-10}$
CVD mortality	1.38<br>[0.72, 2.61]<br>0.3291	1.13<br>[0.58, 2.22]<br>0.7246	1.32<br>[0.67, 2.62]<br>0.4251	3.14*<br>[2.06, 4.79]<br>9.75x10-8	2.27*<br>[1.48, 3.48]<br>1.69x10-4	1.24<br>[0.74, 2.07]<br>0.4076	2.54*<br>[1.81, 3.55]<br>6.64x10-8	2.08*<br>[1.48, 2.94]<br>3.05x10-5	1.46<br>[0.99, 2.16]<br>0.0569
All-cause mortality	1.62*<br>[1.24, 2.11]<br>$4.34\text{x}10^{-4}$	1.35<br>[1.02, 1.78]<br>0.0353	1.41*<br>[1.07, 1.87]<br>0.0162	1.72*<br>[1.36, 2.18]<br>$6.06\text{x}10^{-6}$	1.35*<br>[1.06, 1.71]<br>0.0140	1.14<br>[0.88, 1.47]<br>0.3289	1.43*<br>[1.20, 1.70]<br>$5.56\text{x}10^{-5}$	1.25*<br>[1.04, 1.49]<br>0.0144	1.12<br>[0.93, 1.36]<br>0.2268

Supplementary Table 7 footnote: Entries are hazard ratios for each disease associated with each volume asymmetry type, with 95% confidence intervals and p-values from Cox proportional hazard regression models. Model 1= crude association, Model 2 includes adjustment by age, sex, systolic blood pressure, body mass index, smoking, Townsend deprivation index, hypertension, high cholesterol, diabetes. Model 3 includes Model 2 covariates plus left ventricular mass index, right ventricular ejection fraction and left ventricular ejection fraction. Two-tailed significance was adjusted for multiple testing with a false discovery rate of 5%. (**back to Results**)
